# Transcatheter Aortic Valve Implantation in Cardiogenic Shock: Current Evidence, Clinical Challenges, and Future Directions

**DOI:** 10.3390/jcm14155398

**Published:** 2025-07-31

**Authors:** Grigoris V. Karamasis, Christos Kourek, Dimitrios Alexopoulos, John Parissis

**Affiliations:** 1 Cardiology Department, Attikon University Hospital, National and Kapodistrian University of Athens Medical School, Rimini 1, Chaidari, 12462 Athens, Greece; chris.kourek.92@gmail.com; 2 7th Department of Cardiology, Hygeia Hospital, 15123 Athens, Greece; 3 Heart Failure Unit and University Clinic of Emergency Medicine, Attikon University Hospital, National and Kapodistrian University of Athens Medical School, Rimini 1, Chaidari, 12462 Athens, Greece

**Keywords:** transcatheter aortic valve implantation (TAVI), cardiogenic shock, heart failure, aortic stenosis

## Abstract

Cardiogenic shock (CS) in the setting of severe aortic stenosis (AS) presents a critical and high-risk scenario with limited therapeutic options and poor prognosis. Transcatheter aortic valve implantation (TAVI), initially reserved for inoperable or high-risk surgical candidates, is increasingly being considered in patients with CS due to improvements in device technology, operator experience, and supportive care. This review synthesizes current evidence from large registries, observational studies, and meta-analyses that support the feasibility, safety, and potential survival benefit of urgent or emergent TAVI in selected CS patients. Procedural success is high, and early intervention appears to confer improved short-term and mid-term outcomes compared to balloon aortic valvuloplasty or medical therapy alone. Critical factors influencing prognosis include lactate levels, left ventricular ejection fraction, renal function, and timing of intervention. The absence of formal guidelines, logistical constraints, and ethical concerns complicate decision-making in this unstable population. A multidisciplinary Heart Team/Shock Team approach is essential to identify appropriate candidates, manage procedural risk, and guide post-intervention care. Further studies and the development of TAVI-specific risk models in CS are anticipated to refine patient selection and therapeutic strategies. TAVI may represent a transformative option for stabilizing hemodynamics and improving outcomes in this otherwise high-mortality group.

## 1. Introduction

Cardiogenic shock (CS) is a critical state of circulatory failure resulting from inadequate cardiac output to meet the metabolic demands of the body, leading to tissue hypoperfusion and end-organ dysfunction [1,2]. It is most commonly triggered by acute myocardial infarction (MI), although it may also arise from other etiologies such as decompensated heart failure (HF), myocarditis, valvular disease or malignant arrhythmias [3,4,5].

Aortic stenosis (AS) is a common and progressive valvular heart disease, particularly prevalent among elderly populations [6,7]. Its prevalence is >2% in patients aged >60 years, while the 2-year mortality rate of severe AS is approximately 50% when symptomatic [8,9]. It is primarily caused by age-related calcific degeneration of the aortic valve, leading to restricted valve opening, increased left ventricular (LV) outflow resistance, and eventual pressure overload [9]. In older adults, AS often coexists with multiple comorbidities, which can complicate management.

Transcatheter aortic valve implantation (TAVI) has emerged as a transformative treatment for patients with severe AS. Unlike traditional surgical aortic valve replacement (SAVR), TAVI is a minimally invasive procedure that delivers a bioprosthetic valve via catheter, typically through the femoral artery. Initially reserved for inoperable or high-risk patients, TAVI has since demonstrated comparable or superior outcomes to SAVR in intermediate [10,11] and even low-risk populations [12,13] in several multicenter randomized controlled trials. Its less invasive nature results in reduced perioperative complications, shorter hospital stays and faster recovery, making it an increasingly preferred option for managing severe AS in a big spectrum of appropriately selected patients [14,15].

The use of TAVI is expanding beyond stable patients to include those presenting with hemodynamic instability or CS, reflecting a growing intersection between advanced valve therapy and critical care. Although traditionally considered high-risk or unsuitable candidates for intervention, selected unstable patients with severe AS have shown improved outcomes when treated with urgent or emergent TAVI [16,17,18,19]. Emerging evidence suggests that timely valve replacement in this setting may help restore hemodynamic stability, reduce reliance on vasopressors or mechanical support, and improve survival [18]. However, this evolving application poses unique clinical challenges and underscores the need for careful patient selection and multidisciplinary management.

The aim of this review is to provide an updated and comprehensive overview of the current evidence regarding the use of TAVI in high-risk patients presenting with CS, including clinical outcomes, procedural considerations, selection criteria, as well as challenges associated with performing TAVI in hemodynamically unstable settings. Given the evolving nature of the evidence and the heterogeneity of published studies in this field, we present a narrative review which integrates the most recent real-world registry data with a clinically oriented discussion of patient selection, timing, and outcomes. Furthermore, it provides a forward-looking perspective on future directions, including the need for innovative study designs and a critical appraisal of the evolving role of multidisciplinary Shock/Heart teams.

## 2. Pathophysiology of Cardiogenic Shock in Aortic Stenosis

AS typically develops gradually over many years, beginning with a phase of subclinical inflammation. This early stage leads to fibrotic changes and thickening of the valve leaflets, eventually progressing to significant calcification. The inflammatory process may be triggered by endothelial injury caused by abnormal blood flow across a normal tricuspid valve or a congenitally abnormal valve, such as a bicuspid valve, or it may result from chronic inflammatory conditions like rheumatic heart disease [20]. Regardless of the underlying cause, most cases of AS in adults are marked by extensive calcification by the time symptoms appear. As calcification advances, the valve leaflets become increasingly immobile, the valve opening narrows, and the obstruction to blood flow from the left ventricle to the aorta worsens, ultimately impairing cardiac output [20].

In the setting of severe AS, progressive obstruction to LV outflow, in combination with a critical reduction in cardiac output, results to the development of CS [20,21,22]. The stenotic valve imposes a fixed afterload on the left ventricle, forcing the heart to generate higher pressures to maintain forward flow. Over time, this results in concentric LV hypertrophy, which initially compensates for the pressure overload but eventually leads to diastolic dysfunction, reduced stroke volume, and impaired myocardial perfusion [20,21]. Pulmonary hypertension prevalence ranges between 30% and 37% in severe AS and occurs due to the LV diastolic dysfunction [23]. As the disease progresses, the left ventricle may fail to maintain adequate output, especially during periods of increased demand or decompensation, triggering systemic hypoperfusion and multiorgan dysfunction characteristic of CS [20,21]. Contributing factors such as atrial fibrillation, hypotension, acute coronary syndrome, or volume overload can further destabilize hemodynamics. The interplay between reduced preload, impaired contractility, and elevated afterload creates a vicious cycle that culminates in the collapse of circulatory function if not promptly addressed [24]. As the AS becomes more severe, patients present significant hemodynamic consequences such as exertional dyspnea, angina, or syncope [20]. In advanced stages, the ventricle may decompensate, leading to reduced ejection fraction, elevated left atrial and pulmonary pressures, and ultimately congestive HF [20]. These changes set the stage for hemodynamic collapse and may precipitate CS if left untreated, particularly in the presence of triggering factors like arrhythmias, infections, or other stressors.

**Figure 1:**
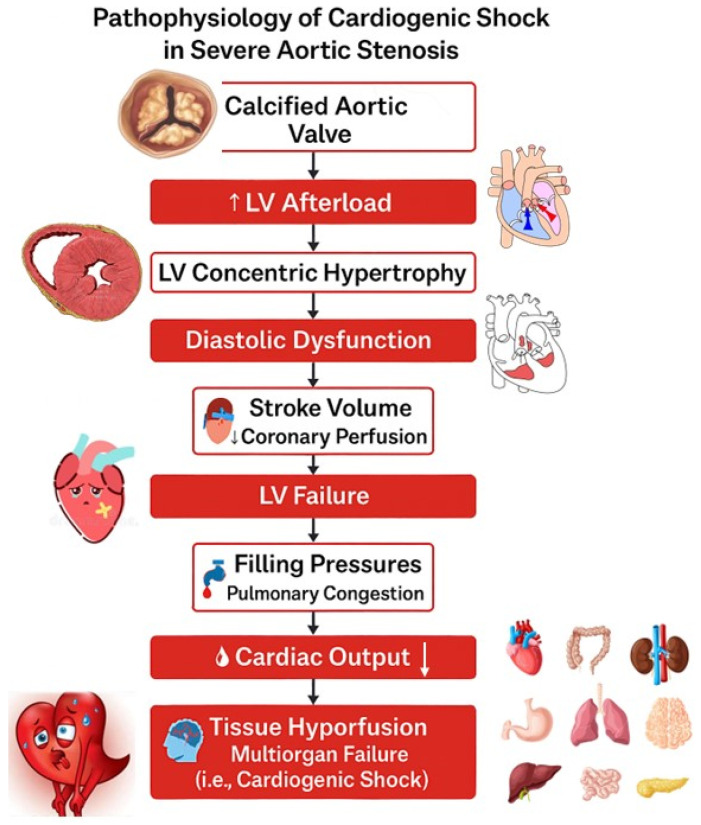
A schematic representation of the pathophysiological progression from severe aortic stenosis to cardiogenic shock. LV, left ventricular; ↓, decrease; ↑, increase.

LV dysfunction plays a central role in the pathophysiology of AS, particularly in the progression to symptomatic HF and CS. In AS, the left ventricle is subjected to a chronically increased afterload due to the narrowed valve orifice, which impedes forward blood flow [22,23,25]. Initially, the heart compensates by developing concentric hypertrophy to preserve systolic function. However, over time, this adaptation leads to impaired ventricular compliance, reduced diastolic filling, and eventually diminished contractile performance [22,23,25]. The condition known as afterload mismatch arises when the ventricular contractility is insufficient to overcome the elevated resistance imposed by the stenotic valve, resulting in a sharp decline in stroke volume and cardiac output [22]. This mismatch becomes particularly critical in patients with reduced LV ejection fraction, where the weakened myocardium is no longer able to generate the pressures needed to maintain forward flow [26]. The resulting decrease in systemic perfusion and increase in intracardiac filling pressures contribute to pulmonary congestion, systemic hypoperfusion, and the clinical manifestations of HF or CS, requiring immediate hemodynamic support (Figure 1) [26,27].

## 3. Clinical Evidence of TAVI in Cardiogenic Shock

### 3.1. Methodology

We performed a comprehensive narrative review using a structured search of the PubMed/MEDLINE, EMBASE, and Cochrane Library databases up to May 2025. Search terms included combinations of “transcatheter aortic valve implantation” or “transcatheter aortic valve replacement” or “TAVI” or “TAVR” and “cardiogenic shock” and “aortic stenosis”. We included original studies, meta-analyses, and large registries reporting on TAVI in patients with CS, without language restrictions. Case reports, editorials, and studies not involving human subjects were excluded. References of included articles were also manually screened to identify additional relevant publications. We included studies reporting on procedural outcomes, short-term (in-hospital or 30-day), and longer-term (up to 1 year) mortality. Case reports, editorials, and studies that did not specify outcomes in the context of CS were excluded.

### 3.2. Study and Patient Characteristics

The included studies were primarily observational in design and comprised both single-center and multicenter cohorts, with sample sizes ranging from fewer than 100 to over 10,000 patients. Most studies were retrospective; however, some prospective cohorts and registry analyses were also included. Mean patient age across studies ranged from 78 to 85 years, with a predominance of female patients in some cohorts. Patients undergoing TAVI in the setting of CS generally had higher surgical risk scores (STS and EuroSCORE), worse LV function, and a greater burden of comorbidities compared to those undergoing elective TAVI.

### 3.3. Clinical Outcomes

Effective management of AS in hemodynamically unstable patients with CS requires immediate hemodynamic support and interventional approach of AS treatment. TAVI was initially developed back in 2002 by Alain Cribier as a less invasive alternative to SAVR for patients with severe AS who were deemed inoperable or at high surgical risk due to advanced age, frailty, or multiple comorbidities [28]. Early in its adoption, TAVI was considered unsuitable for hemodynamically unstable patients, including those in CS, due to concerns over procedural complexity, lack of robust data, and the potential for poor outcomes. Traditional contraindications included severe left ventricular dysfunction, unstable hemodynamics, and the need for urgent intervention in critically ill patients, as these conditions were thought to increase peri-procedural risk [29]. However, advancements in valve technology, procedural techniques, and perioperative care have significantly broadened the scope of TAVI. New-generation devices offer improved deliverability, repositionability, and sealing, while operator experience and multidisciplinary heart team approaches have enhanced patient selection and procedural planning. As a result, TAVI is increasingly being considered for appropriately selected unstable patients in emergent settings, including those with CS, offering a potentially life-saving option where previously no feasible treatment existed [30]. Many recent observational studies have explored feasibility, safety and clinical outcomes of TAVI in high-risk patients presenting with CS [19,31,32,33,34,35,36,37,38,39,40,41,42].

#### 3.3.1. Evidence from Large Registries

Evidence from large national and international registries reinforces the feasibility of TAVI in patients with CS, albeit with high associated mortality. Large registry data, including the Society of Thoracic Surgeons/American College of Cardiology Transcatheter Valve Therapy (STS/ACC TVT) Registry [43], and the German Aortic Valve Registry (GARY) [44], have provided valuable insights into the real-world outcomes of TAVI in hemodynamically unstable patients. These registries suggest that although in-hospital complication rates and mortality remain elevated in this subgroup, selected patients can derive meaningful survival benefit from early intervention.

A recent large-scale, real-world study by Goel et al. [42], used data from the STS/ACC TVT Registry [43] (a comprehensive national database established to monitor outcomes and procedural trends of TAVI in the United States) in order to evaluate the safety and outcomes of TAVI in patients with severe AS presenting with CS. The analysis included over 309,000 patients who underwent TAVI with a balloon-expandable valve (SAPIEN 3 or SAPIEN 3 Ultra, Edwards Lifesciences, Irvine, CA, USA) between June 2015 and September 2022. Of these, 5006 (1.6%) met the study’s criteria for CS based on registry coding, use of inotropes or mechanical circulatory support (MCS), or cardiac arrest within 24 h before the procedure. Procedural success was remarkably high, as implantation was successful in nearly 98% of CS cases, and technical success per VARC-3 criteria was achieved in 94.5%. Compared to a propensity-matched control group without CS, patients with CS had significantly higher in-hospital (9.9% vs. 2.7%), 30-day (12.9% vs. 4.9%), and 1-year mortality rates (29.7% vs. 22.6%). However, among patients who survived the first 30 days, 1-year mortality rates were similar between the two groups, highlighting a potential survival benefit if patients can be stabilized beyond the acute phase. The study also found notable improvements in functional status and quality of life among survivors, with 89% of CS patients classified as NYHA I/II and a significant increase in Kansas City Cardiomyopathy Questionnaire (KCCQ) scores at one year. Predictors of increased 1-year mortality included advanced age, end-stage renal disease, peripheral artery disease, immunocompromised status, and prior implantable cardioverter-defibrillator placement. Moreover, lower albumin and hemoglobin levels, as well as reduced aortic valve gradients, were also associated with worse outcomes. Importantly, early TAVI within five days of admission was linked to lower short- and long-term mortality compared to delayed intervention. This study is important as it consists of the largest dataset of patients with severe AS and hemodynamic instability treated with TAVI. However, its results should be interpreted in the context of its limitations. Its observational retrospective design raises the possibility of significant selection bias with the most severely ill patients being excluded from interventional treatment. Evermore, the used definition of CS did not incorporate hemodynamic data or data on markers of tissue hypoperfusion. This data is an integral part of contemporary CS definitions and severity staging systems. Consequently, there is a question whether the population in the CS group was in a truly critical state [44]. Nevertheless, the data suggest that, in selected patients, prompt TAVI may help reverse hemodynamic compromise and improve survival even in the context of CS.

An older, large registry from Europe, the Gary Registry [45], provided one of the most comprehensive real-world datasets on aortic valve interventions, including TAVI in high-risk populations. In the 2011 registry cohort, which included over 13,800 patients, a significant subset of individuals undergoing TAVI presented in critical condition, including those in CS. Specifically, 3.9% of patients treated with transvascular TAVI (TV-TAVI) and 1.9% of those undergoing transapical TAVI (TA-TAVI) were in CS within 48 h prior to the procedure. These patients also showed higher rates of inotropic support and mechanical ventilation at the time of intervention, underscoring their hemodynamic instability. The in-hospital mortality for TV-TAVI was 5.1%, while TA-TAVI had a higher rate of 7.7%. These acceptable mortality rates suggest that TAVI could be a viable option even in patients presenting with CS, especially when performed in experienced centers. Procedural success was high, with the vast majority of patients achieving adequate valve function and only low rates of significant aortic regurgitation or conversion to open surgery. Importantly, patients in CS undergoing TAVI were typically older, had higher EuroSCORE (mean > 25), and suffered from more comorbidities such as pulmonary hypertension, renal dysfunction, and reduced ejection fraction. These characteristics further highlight the complexity of managing this subgroup. The registry also emphasized the need for timely and multidisciplinary decision-making, often involving dedicated heart teams. In summary, TAVI in CS seemed to be both feasible and potentially life-saving through the GARY Registry, highlighting that selected patients with severe AS and hemodynamic collapse may benefit from urgent TAVI.

Another European multicenter registry [35], evaluated the safety and feasibility of TAVI in 51 critically ill patients with severe aortic valve disease and CS. The majority were elderly and high-risk, with extensive comorbidities and requiring inotropic or mechanical support. TAVI was performed emergently, predominantly via transfemoral access, and achieved a high procedural success rate of 94%. Thirty-day mortality was 11.8%, and 1-year survival reached 74.3%, with most survivors in good functional status. Complications included acute kidney injury and vascular events, though severe paravalvular leak and procedural deaths were rare. These findings supported TAVI as a viable option for selected patients in CS when performed in experienced centers.

Finally, a large retrospective analysis by Llah et al. [46], used data from the National Inpatient Sample to compare outcomes of TAVI and BAV in over 11,000 hospitalizations involving patients with severe AS complicated by CS between 2016 and 2020, showing that direct TAVI may offer a more effective and durable treatment option than rescue BAV. Following propensity score matching, 3485 cases from each group were analyzed. TAVI was significantly associated with improved in-hospital outcomes compared to BAV. Specifically, the rates of primary outcomes events [36.8% vs. 56.8%, aOR (95%CI) = 0.38 (0.30–0.47)], all-cause in-hospital deaths [17.8% vs. 38.9%], and myocardial infraction [12.3% vs. 32.4%, aOR (95%CI) = 0.29 (0.22–0.39)] were lower in the TAVI group compared to BAV. However, TAVI was linked to a slightly increased risk of stroke [6.17% vs. 3.44%, aOR (95%CI) = 1.84 (1.08–3.21)] and pacemaker implantation [11.9% vs. 6.03%, aOR (95%CI) = 2.10 (1.41–3.18)].

These large-scale data sources highlight that while mortality remains elevated, particularly in shock patients, TAVI remains a technically reliable and increasingly adopted strategy in contemporary real-world practice. Importantly, outcomes are strongly influenced by patient selection, timing, and institutional experience.

#### 3.3.2. Evidence from Observational Studies and Small Cohorts

Additionally, smaller observational cohorts have confirmed that timely TAVI in the context of CS may lead to hemodynamic stabilization and improved short- to mid-term outcomes (Table 1). Piriou et al. [39], assessed 38 critically ill patients with severe aortic valve disease and CS who underwent rescue TAVI. The procedure had no intraprocedural deaths, with 30-day and 1-year mortality rates of 7.9% and 21.1%, respectively. Complications included acute kidney injury, left bundle branch block, and pacemaker implantation, but functional and echocardiographic outcomes were favorable. Authors showed that rescue TAVI in patients with CS is a feasible and generally safe intervention with encouraging short- and mid-term survival rates.

During the last decade, observational studies have investigated urgent balloon aortic valvuloplasty (BAV) in patients with CS related to severe AS showing favorable outcomes in the acute setting [47,48]. However, eventually mid and long-term outcomes were not altered, and critically ill patients continued having a dismal prognosis [19,47,48]. Other studies compared TAVI and BAV seeking to establish superiority of one or the other method in the acute setting. A retrospective cohort study by Bongiovanni D et al. [34], evaluated 141 elderly high-risk patients with severe decompensated AS, including those in CS, to compare outcomes of emergency TAVI (eTAVI) versus emergency BAV (eBAV) followed by elective TAVI. Of the cohort, 23 patients underwent eTAVI and 118 received eBAV. The 30-day all-cause mortality was high in both groups, 23.8% for eTAVI and 33.0% for eBAV, but not significantly different. Immediate procedural mortality was lower with eTAVI (8.7%) compared to eBAV (20.3%), and eTAVI also resulted in fewer cases of postprocedural aortic regurgitation. However, eTAVI was associated with a higher rate of stroke (8.7% vs. 0%, respectively) and major vascular complications (17.4% vs. 3.4%, respectively). Interestingly, elective TAVI following eBAV did not offer a clear survival advantage and carried a higher than expected 30-day mortality (21.9%). These results suggest that while both emergency strategies carry substantial risk, eTAVI may offer a viable direct approach in select unstable patients. Similar findings were shown a few years later by Ali et al. [49]. At 30 days, nearly 98% of TAVI patients were alive, compared to 88.5% in the BAV group. The gap widened by one year, with 88.5% survival in the TAVI cohort versus only 44.2% in those treated with BAV.

Another study a few years ago analyzed nationwide data to compare hospital readmissions between urgent TAVI and BAV in patients with severe AS complicated by CS or decompensated HF [50]. Urgent TAVI was associated with lower 30- and 90-day readmission rates [15.4 vs. 22.5%, (aHR): 0.92 (0.90–0.95) *p* < 0.001] and reduced 30-day readmission due to CV cause and HF (aHR, 0.93: *p* < 0.001 and aHR, 0.98: *p* = 0.040, respectively), although it carried a three times increased risk of gastrointestinal bleeding [aHR, 3.00: 95%CI (1.23–7.33), *p* = 0.016] within 30 days. Overall, the findings suggest that urgent TAVI may offer better clinical stability and fewer hospital readmissions compared to BAV, supporting its role as a preferred approach in selected high-risk patients.

**Figure 2:**
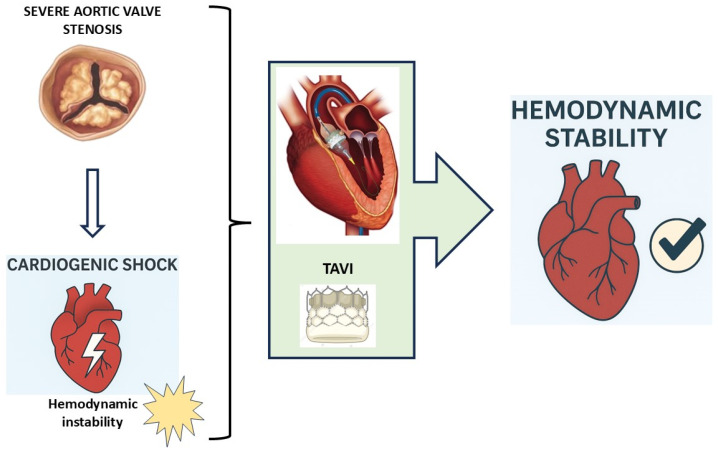
A conceptual diagram illustrating the therapeutic role of transcatheter aortic valve implantation in restoring hemodynamic stability in cardiogenic shock. TAVI, transcatheter aortic valve implantation.

Compared to BAV and/or medical medication, TAVI seems to have beneficial effects on the outcomes of patients with severe AS and CS. A recent single-center retrospective cohort study from the Cleveland Clinic [19], examined 2754 patients admitted to the cardiac intensive care unit with CS between 2010 and 2021, identifying 199 (7%) with CS due to severe AS. These patients faced significantly higher short-term mortality than those with CS from other causes. The study compared outcomes across four treatment strategies: medical management, BAV, SAVR, and TAVI. Among patients undergoing transcatheter interventions, 24 received urgent TAVI, while 46 underwent BAV. TAVI was associated with notably better outcomes than BAV and medical therapy. The 30-day mortality rate for TAVI was just 4%, compared to 26% for BAV and 50% for medically managed patients. At one year, TAVI and SAVR showed similar survival benefits, both substantially outperforming BAV and medical management. Importantly, the study highlighted that while BAV can temporarily stabilize critically ill patients, its survival advantage rapidly declines without timely transition to definitive valve replacement, ideally within 30 to 90 days. Multivariable analysis confirmed that both TAVI and SAVR independently predicted lower mortality, while mechanical ventilation was associated with worse outcomes. Overall, this study supports the use of TAVI as a safe and effective treatment option in selected patients with severe AS and CS (Figure 2). It emphasizes the need for timely definitive valve replacement, suggesting that TAVI should be considered early in the treatment course for this high-risk population. All the previous studies should be considered under the limitations set by their observational and commonly retrospective design. It is inevitable that selection bias would have happened and patients with more severe diseases or comorbidities would have been excluded from resource-demanding interventions like TAVI.

Procedural success rates for TAVI in the setting of CS remain encouragingly high despite the critically ill nature of these patients. Across the reviewed studies, device success was consistently reported to be over 90%. These findings suggest that TAVI remains a technically feasible and effective treatment even in the most unstable hemodynamic conditions, provided the procedure is performed in experienced centers. Procedural failure, while uncommon, is more often linked to patient-related factors such as low valve gradients, severe LV dysfunction, or vascular access limitations rather than device-related issues.

A summary of studies regarding data and outcomes after TAVI in patients with CS, is demonstrated in Table 1. Table 2 presents more details regarding the intervention, the main outcomes and the conclusions of each study.

The prognosis of patients undergoing TAVI in the context of cardiogenic shock (CS) is markedly worse than that of elective cases. However, recent data suggest that early intervention may still offer survival benefits, particularly in experienced centers.

A meta-analysis by Wernly et al. [51] included 19 observational studies with over 2000 patients undergoing TAVI in the setting of CS and reported a pooled 30-day mortality rate of 22.6%, with a 1-year mortality of 44.9%. Procedural success was high (>90%), while stroke and major bleeding occurred in 4.0% and 5.8%, respectively.

Short-term mortality varies widely based on patient selection, timing and institutional expertise. In published studies, 30-day mortality after TAVI in patients with CS ranges between 12% and 33.3% [42,52]. Importantly, in-hospital survival is strongly influenced by rapid stabilization and early definitive intervention, with data suggesting that patients who survive the initial hospitalization or 30-day window tend to have favorable subsequent outcomes [32,42].

Several clinical and biochemical markers have been identified as predictors of poor prognosis following TAVI in CS. Elevated serum lactate [53], reduced LV ejection fraction [54], advanced renal dysfunction [55,56], hypoalbuminemia [57], and need for mechanical ventilation at the time of procedure are consistently associated with increased mortality [58]. Elevated lactate levels, in particular, reflect systemic hypoperfusion and correlate with multiorgan dysfunction [59], while severely decreased LVEF signals limited cardiac reserve [60]. Nonetheless, a subset of patients experiences meaningful recovery of ventricular function after relief of the valvular obstruction, often within days to weeks post-TAVI [61]. Studies have reported significant improvements in LVEF and cardiac output, especially in patients without irreversible myocardial damage. These hemodynamic gains frequently translate into improved functional status, with many survivors achieving NYHA class I or II at follow-up [42]. Mid-term survival rates, particularly beyond one year, can approach those of stable TAVI cohorts if patients recover from the acute phase, supporting the therapeutic value of TAVI in appropriately selected patients with CS [62,63].

## 4. Practical Considerations in TAVI Procedure During Cardiogenic Shock

### 4.1. Role of Balloon Aortic Valvuloplasty and Contraindications to TAVI in Cardiogenic Shock

In select patients with CS due to severe AS, BAV may serve as a bridge therapy to stabilize hemodynamics before definitive valve replacement. While BAV has largely been abandoned as a stand-alone therapy due to high restenosis rates, it may offer temporary relief in unstable patients where immediate TAVI is not feasible due to anatomical, logistical, or patient-related constraints. In the setting of acute decompensation, BAV may improve cardiac output and reduce afterload, potentially improving outcomes when followed by staged TAVI. However, observational studies have shown that BAV alone is associated with higher short- and mid-term mortality compared to definitive TAVI, reinforcing its role primarily as a temporizing measure [34,49,50].

TAVI, although feasible in CS, is not suitable for all patients. Contraindications include severe aortic annular calcification with high risk of rupture, unfavorable iliofemoral anatomy for transfemoral access with no alternative route available, active endocarditis, or inability to achieve coronary access post-implantation in unstable patients [52]. Additionally, patients with very low transvalvular gradients, profound LV dysfunction with limited contractile reserve, or severe multiorgan failure may derive limited benefit and experience poor outcomes despite successful valve deployment [52]. In such scenarios, the risks may outweigh the benefits, and conservative or palliative strategies may need to be considered.

### 4.2. Timing of Intervention

Timing of TAVI remains a significant consideration in patients severe AS complicated by CS, playing a critical role in determining outcomes. Emergent TAVI, defined as valve implantation performed during the index hospitalization for CS, is increasingly being adopted as a life-saving intervention in hemodynamically unstable patients [64]. Several studies, including large registry data, have demonstrated that early definitive intervention with TAVI leads to improved short-term survival compared to medical management or temporizing measures such as BAV [19,42]. Urgent TAVI, typically performed within days of stabilization, may also offer favorable outcomes, especially when preceded by brief hemodynamic support to allow for patient optimization [65].

In contrast, a staged approach, often starting with BAV followed by delayed TAVI, has shown mixed results. While it may benefit selected patients who are too unstable for immediate valve implantation, outcomes worsen significantly if definitive treatment is delayed beyond 60–90 days [66]. Thus, the consensus emerging from contemporary literature supports early definitive valve replacement, preferably during the same hospitalization, for patients with CS and severe AS, provided anatomical and clinical factors permit [19,67]. Nevertheless, BAV has a number of advantages like smaller insertion profile, lower risk of vascular complications, higher availability and lower cost and could be considered as a bridge to TAVI in selected patients [52].

### 4.3. Valve Choice and Access Point

Valve choice and access are also significant considerations in TAVI procedure in patients with severe AS and CS that impact safety and success. Balloon-expandable and self-expanding valves are both utilized, with device choice often guided by anatomical characteristics such as annular size, calcium burden, and proximity to coronary ostia [68,69]. Balloon-expandable valves may offer greater positional accuracy [70], which is valuable in unstable patients, while self-expanding valves can be advantageous in smaller annuli or in patients with borderline hemodynamics due to their gradual deployment and repositionability [71,72]. In general terms, devices anticipating the best outcomes with least hemodynamic compromise during deployment should be preferred [52]. On the other hand, since there is no strong evidence supporting a specific device, operators should choose the valve they are more familiar with [52]. Vascular access should be transfemoral, given its minimally invasive nature and association with faster recovery [52,73]. Alternative approaches such as trans-axillary or transcarotid are theoretically feasible but they should only be used as last resort in highly selected patients. The feasibility of rapid access and deployment is especially important in CS, where procedural efficiency can influence outcomes. Contemporary studies and registries highlight that successful transfemoral TAVI in CS patients is associated with lower mortality and complication rates [15,74].

### 4.4. Use of Mechanical Circulatory Support and Anesthetic Approach

The potential use of mechanical circulatory support (MCS) devices and the choice of anesthetic approach are critical components of peri-procedural management. MCS options such as intra-aortic balloon pump (IABP), Impella, and veno-arterial extracorporeal membrane oxygenation (VA-ECMO) have been used to stabilize hemodynamics, maintain end-organ perfusion, and bridge patients to definitive valve intervention [75,76,77,78]. IABP, though with low complication risk provides only modest support and its actual benefit is questionable [75,79,80]. Impella offers direct left ventricular unloading and a reasonable increase in cardiac output and has been used as a bailout option in patients with CS and severe AS [81]. However, in a single-center experience where it was used as a bridge to TAVI along with BAV, 30-day mortality was as high as 45% [82]. VA-ECMO provides the highest hemodynamic and respiratory support, especially in cases of multiorgan failure or profound shock, though it carries a higher risk of bleeding, vascular injury, and limb ischemia [83]. Since there is limited published evidence, use and selection of MCS should be carefully studied and individualized based on the extent of hemodynamic compromise, ventricular function, and institutional expertise. In patients with CS due to severe AS, MCS may also be necessary when concomitant high-risk coronary interventions or arrhythmia ablation are required. In such complex revascularization scenarios, such as left main disease, multi-vessel PCI, or chronic total occlusion, devices like Impella or ECMO provide valuable hemodynamic support to maintain end-organ perfusion and procedural stability. As highlighted in recent reports [84], combining MCS with early revascularization strategies may improve survival in select high-risk patients. Tailoring the choice and timing of MCS requires a multidisciplinary approach, considering both the severity of myocardial dysfunction and the anticipated procedural complexity.

MCS can provide critical hemodynamic stabilization in patients with profound CS undergoing or awaiting TAVI, by maintaining cardiac output and organ perfusion. However, the timing and selection of MCS remain controversial. Early deployment may prevent end-organ damage and improve procedural safety, but it also carries risks of vascular complications, bleeding, and device-related issues.

Observational studies suggest that the need for MCS is an independent predictor of mortality following TAVI in CS, likely reflecting the severity of underlying cardiac dysfunction. Some centers advocate for a prophylactic approach in unstable patients, while others limit MCS to rescue therapy after hemodynamic collapse. At present, no randomized data exist to guide practice, and decisions are often individualized based on center expertise, resource availability, and patient comorbidities. Nonetheless, integration of MCS into TAVI care pathways is increasingly relevant and should be considered in early treatment planning for patients with severe aortic stenosis and cardiogenic shock.

Anesthetic strategy also plays a pivotal role; while general anesthesia was traditionally in the early days of TAVI, local anesthesia or conscious sedation are the contemporary standard of care [85]. This approach minimizes hemodynamic fluctuations, reduces the need for vasoactive agents, and allows for faster post-procedural recovery. In a recent RCT, conscious sedation showed better hemodynamic stability and a strong safety profile [85]. However, with a hemodynamically unstable patient with CS, general anesthesia could be the only option for procedural feasibility. In any case, as shown the prementioned, a significant number of patients with severe AS and CS require mechanical ventilation and are intubated even before any invasive procedure.

### 4.5. Imaging in TAVI and Existing Coronary Artery Disease

Imaging plays a critical role in ensuring the safety and efficacy of contemporary TAVI procedures, with multi-detector computed tomography (CT) now considered the gold standard for pre-procedural planning [86,87,88]. TAVI CT allows accurate assessment of vascular access, annular dimensions, valve morphology, and spatial relationships, all of which are essential for selecting the appropriate valve size and minimizing complications such as annular rupture or paravalvular leak [86]. However, in the setting of CS, performing a full CT protocol can be challenging or even unfeasible due to hemodynamic instability, renal dysfunction, or the inability to transfer the patient safely to the scanner [89]. In such situations, transesophageal echocardiography (TOE) can serve as an alternative tool, particularly for annular sizing and functional assessment of the valve apparatus, although it lacks the comprehensive anatomical detail provided by CT [90]. The inability to perform CT imaging in emergent settings may partially explain the increased procedural risks and underscores the importance of individualized decision-making when proceeding with TAVI under suboptimal imaging conditions.

Another important consideration in the setting of CS and severe AS is the management of coexisting CAD. ACS can act as a precipitating factor for decompensation and the onset of shock in patients with underlying AS, further complicating both diagnosis and therapeutic strategy [91,92]. In such cases, emergent coronary angiography is often warranted to identify culprit lesions that may require revascularization prior to or in conjunction with TAVI [91,93,94]. However, even in the absence of ACS, the presence of stable CAD raises questions about timing and prioritization; whether to stage revascularization, perform it concomitantly with TAVI, or defer entirely [92]. These decisions must be individualized, taking into account the burden and location of CAD, the feasibility of complete revascularization, hemodynamic status, and anticipated recovery potential. The lack of randomized data in this context underscores the need for Heart Team or Shock Team involvement to guide optimal sequencing and integration of therapies.

### 4.6. Individualized Approach via the Heart Team/Shock Team

Given the high complexity and time-sensitive nature of decision-making in patients with severe AS and CS, the early involvement of a multidisciplinary Heart Team is of paramount importance. This team typically includes interventional cardiologists, cardiac surgeons, HF specialists, anesthesiologists, and imaging experts, each contributing their perspective to assess procedural feasibility, risk, and potential benefit [52]. In the context of CS, timely collaboration becomes even more critical, as delays in intervention may worsen outcomes. A structured team approach ensures thorough evaluation of patient status, appropriateness of mechanical support, anatomical considerations, and post-procedural care [52]. Moreover, the Heart Team facilitates balanced decision-making in ethically sensitive scenarios, aligning treatment plans with the patient’s values and goals of care. As TAVI expands into more complex and unstable populations, institutional protocols that support rapid, coordinated Heart Team discussions are crucial to ensure responsible, patient-centered care. It is important to emphasize that its engagement is not optional but rather mandatory in balancing potential benefit against futility [52]. This collaborative approach ensures that all aspects of the patient’s clinical condition, comorbidities, anatomical suitability, and likelihood of recovery are thoroughly evaluated before proceeding with an emergent intervention such as TAVI. Factors that promote utility of TAVI in CS patients are i. young age, ii. lack of extracardiac comorbidities, iii. high transvalvular gradient, iv. the early stage of CS, v. the high experience of TAVI centers, and vi. the feasibility of transfemoral access [52]. On the other hand, not every patient is eligible for TAVI during CS. Futility factors include i. older age, ii. severe extracardiac comorbidities unlikely to improve after the intervention, iii. low transvalvular gradient, iv. long-lasting shock symptoms, v. non feasible transfemoral access, and vi. non-experienced TAVI center, without improvement after BAV, and need for transfer to a more experienced center [52].

**Figure 3:**
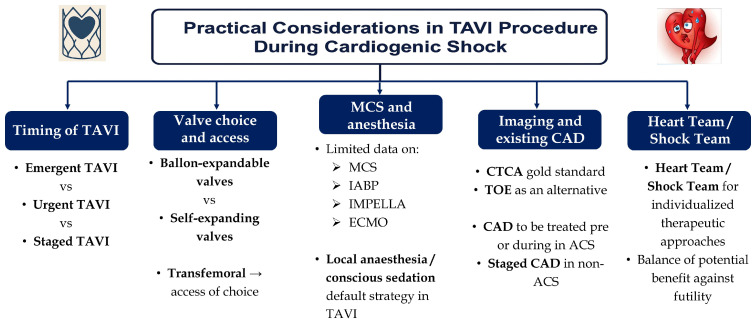
Practical considerations in transcatheter aortic valve implantation procedure during cardiogenic shock. TAVI, transcatheter aortic valve implantation; BAV, balloon aortic valvuloplasty; MCS, mechanical circulatory support; IABP, intra-aortic balloon pump; LV, left ventricle; ECMO, extracorporeal membrane oxygenation; CTCA, computed tomography coronary angiography; TOE, transoesophageal echocardiography; CAD, coronary artery disease; ACS, acute coronary syndrome.

The sum of the most important procedure parameters is demonstrated in Figure 3.

## 5. Challenges and Limitations

Despite the increasing use of TAVI in patients with CS, current international guidelines do not offer specific recommendations for this high-risk subgroup. Most clinical trials that informed existing TAVI guidelines excluded patients in shock or requiring mechanical circulatory support, leaving a significant evidence gap. As a result, the decision to proceed with TAVI in extremis is often based on limited observational data, institutional experience, and clinical judgment rather than standardized protocols. This ambiguity poses ethical challenges, especially when the prognosis is uncertain and the intervention is resource-intensive. Clinicians must weigh the potential for meaningful recovery against the risks of futile care, prolonged suffering, or poor quality of life. The urgency of decision-making in CS further complicates informed consent, as patients may be incapacitated and families must make decisions under emotional distress, highlighting the ethical complexity of offering TAVI in such settings. Furthermore, not all hospitals are equipped to perform urgent TAVI, which may necessitate transferring the patient to a specialized center. Even in institutions where TAVI is available, logistical constraints can delay the ability to perform the procedure promptly [52]. Given the absence of definitive guidelines and the complexity of managing CS in severe AS, multidisciplinary decision-making through a dedicated Heart Team is essential [52]. A new treatment approach besides Heart Team has been discussed during the last years. Shock Team represents a structured, multidisciplinary response model designed to accelerate diagnosis and guide the timely initiation of advanced therapies in patients with CS [95,96]. Given the critical nature of CS, where each passing hour can determine survival and functional recovery, the deployment of a Shock Team ensures rapid evaluation and coordinated decision-making. This urgency is especially vital, as evidence suggests that early implementation of MCS, ideally within the first 48 h, is associated with improved outcomes [95,96]. The importance of initiating the shock management algorithm directly from the Emergency Department, where early hemodynamic assessment and triage can lead to prompt activation of the Shock Team and rapid transfer to definitive care, has already been shown through studies [95]. However, alongside the need for swift action lies the challenge of futility. In patients with advanced shock stages, myocardial and systemic damage may become irreversible, rendering even the most aggressive interventions ineffective. In such cases, the Shock Team plays a crucial role not only in determining candidacy for high-risk interventions like TAVI but also in identifying when such measures are unlikely to provide benefit, thus avoiding unnecessary escalation and focusing instead on palliative or supportive care [95].

The available literature on TAVI in CS remains limited, with most data derived from observational studies, retrospective analyses, or subgroup evaluations from larger registries. This inherently introduces several potential biases. First, survival bias may occur in registries where patients who die before referral or evaluation for TAVI are not captured, thereby overestimating procedural benefit. Second, publication bias may lead to an overrepresentation of favorable outcomes, especially as reports from unsuccessful or complicated cases may be underreported. Third, many of the published series come from high-volume, tertiary care centers with experienced heart teams and immediate access to advanced support technologies (e.g., MCS, surgical backup). Therefore, the external validity of these findings may be limited when applied to lower-volume or non-specialist institutions. Finally, the heterogeneity in definitions of CS, timing of intervention, and reporting of complications limit pooled interpretations and complicates direct comparisons between studies.

## 6. Future Perspectives

As TAVI continues to be adopted in increasingly high-risk populations, including those with severe AS and hemodynamic compromise/CS, there is a growing need for robust clinical evidence to guide management. Although robust evidence from RCTs is essential to guide clinical decision-making, conducting high-quality studies in CS remains extraordinarily challenging. The heterogeneity of clinical presentations, the urgent need for intervention, and ethical concerns around randomizing critically ill patients all pose substantial barriers to trial design and execution. Moreover, difficulty of obtaining consent in emergent settings, rapid patient deterioration, and variable institutional capabilities further complicate recruitment and protocol adherence. As such, despite the clinical urgency and interest, large-scale RCTs dedicated specifically to TAVI in CS populations are extremely challenging due to logistical, ethical, and methodological barriers. However, this should not preclude efforts to generate higher-quality evidence through pragmatic trial designs, adaptive protocols, or international collaborative registries. A call for innovative strategies and stronger evidence generation is urgently needed to support the Heart/Shock Team decision-making process, which currently relies on limited and potentially biased observational data. In this context, real-world data from registries, meta-analyses, and structured observational studies provide the best available insights and form a pragmatic foundation for guiding practice.

Risk stratification and the need for tailored assessment tools are also significant aspects of this syndrome; however, it is challenging due to the dynamic, rapidly evolving nature of the condition and the wide heterogeneity in patient profiles, underlying etiologies, and comorbidities. The complexity of managing TAVI in patients with CS underscores the need for dedicated risk stratification models. Traditional scoring systems, such as the IABP-SHOCK II [97] or SAVE score [98,99], may offer general prognostic guidance but often lack the granularity or timeliness needed for individualized decisions, particularly in the acute phase. Moreover, other scores such as the STS and EuroSCORE II, were not designed to account for the hemodynamic instability, organ dysfunction, or rapid deterioration often seen in CS; however, showing satisfactory discrimination and calibration for predicting 30-day and 1-year mortality in patients undergoing TAVI [100]. As a result, these tools may underestimate procedural risk or fail to capture key prognostic factors unique to this population.

Recent studies have highlighted several predictors of poor outcomes in CS patients undergoing TAVI, including severely reduced LV ejection fraction [101], renal impairment [32,102], and the need for mechanical ventilation or circulatory support [32,102]. Biomarkers and hemodynamic parameters, while helpful, may not fully capture the trajectory of organ dysfunction or the reversibility of shock. In this uncertain landscape, shared decision-making becomes critical. Engaging a multidisciplinary Heart or Shock Team, enables a more nuanced assessment that considers not only clinical risk but also patient values, expected quality of life, and goals of care. This collaborative approach ensures that therapeutic decisions, especially those involving invasive interventions like TAVI or mechanical circulatory support, are made with transparency, alignment with patient preferences when possible, and a balanced view of potential benefit versus futility. The development of validated, TAVI-specific risk scores tailored to critically ill patients could support clinical decision-making, help identify those most likely to benefit from intervention, and guide discussions with patients and families about prognosis.

## 7. Conclusions

TAVI has rapidly evolved from a treatment reserved for inoperable patients with severe AS to a life-saving intervention in those presenting with CS. Contemporary evidence from large-scale observational studies supports the feasibility and potential survival benefit of early or urgent TAVI in this high-risk population. Despite the inherent procedural and hemodynamic challenges, high implantation success rates and favorable outcomes among survivors reinforce its role in selected patients, particularly when performed in experienced centers. Factors such as patient selection, early intervention, and transfemoral access are pivotal to optimizing outcomes, while emerging data highlight the importance of addressing logistical barriers, and refining risk prediction tools. Yet, the absence of formal guideline recommendations and randomized controlled trials leaves clinicians navigating uncertainty in this critically ill group. Large multicenter randomized controlled studies, standardized protocols, and multidisciplinary Heart/Shock team collaboration will be essential to improve decision-making, personalize care, and define the optimal therapeutic window for TAVI in patients with CS.

## Figures and Tables

**Table 1:** Summary of data and clinical outcomes among trials on TAVI in patients with cardiogenic shock.

Authors	Year	Design	Setting	Number of Patients	Mean Age (Years)	Mean LVEF (%)	30-Day Mortality (%)	1-Year Survival (%)
D’Ancona et al. [31]	2012	Prospective	Single center	21	74.5 ± 11.1	26.0 ± 13.1	19.0	46.0
Frerker et al. [32]	2016	Retrospective	Single center	27	78.0 ± 9.0	39.5 ± 15.4	33.3	46.0
Landes et al. [33]	2016	Retrospective	Single center	27	80.1 ± 9.7	Preserved LVEF: 48.1% of patients	3.7	NA
Bongiovanni et al. [34]	2018	Retrospective	Multicenter	23	76.0 ± 11.4	NA	23.8	NA
Kolte et al. [18]	2018	Retrospective	Multicenter	3952	84 (78–88)	53.0 (37.0–60.0)	8.7	70.9
Huang et al. [36]	2019	Retrospective	Single center	26	73.1 ± 13.9	31.8 ±15.3	19.4	61.0
Bandyopadhya et al. [37]	2020	Retrospective	Multicenter	2136	81.4 ± 8.3	NA	TAVI vs. BAV (*p* = 0.29)	NA
Fraccaro et al. [35]	2020	Retrospective	Multicenter	51	75.8 ± 12.9	≤35% in 29.4%	11.8	74.3
Masha et al. [38]	2020	Retrospective	Multicenter	2220	83 (77–87)	53 (33–60)	19.1	65.0
Piriou et al. [39]	2022	Retrospective	Multicenter	38	NA	LVEF < 30% in 78.9%	7.9	78.9
Steffen et al. [40]	2022	Retrospective	Single center	47	81.2 (71.4–86.1)	38.0 (28.5–45.0)	19.1	≈51.1
Castelo et al. [41]	2023	Retrospective	Single center	79	86.9 ± 7.5	45	17.5	NA
Goel et al. [42]	2023	Retrospective	Multicenter	4952	75.6 ± 10.9	39.9 ± 17.6	12.9	70.3
Nair et al. [19]	2024	Retrospective	Single center	24	79.0 (75.5–84.3)	33.5 (22.0–41.3)	4.2	≈81.0

TAVI, transcatheter aortic valve implantation; LVEF, left ventricular ejection fraction; BAV, balloon aortic valvuloplasty; NA, not available.

**Table 2:** Interventions, main clinical outcomes and conclusions among trials on TAVI in patients with cardiogenic shock.

Authors	Intervention	Outcomes	Conclusions
D’Ancona et al. [31]	Patients in CS underwent transapical TAVI	30-day mortality significantly higher in the CS group (19%) vs. non-CS (5%; *p* = 0.02) 1-year survival significantly lower in CS (46%) vs. non-CS (83%; *p* < 0.0001) EuroSCORE determinant for follow-up mortality (odds ratio = 1.02; *p* = 0.04)	TAVI in patients who are in cardiogenic shock is feasible.
Frerker et al. [32]	TAVI in patients with CS due to acutely decompensated AS	30-day mortality 33.3% Predictors of 30-day mortality ✓ baseline cardiac output <3.0 L/min ✓ reduced cardiac power index ✓ impaired renal function ✓ mechanical ventilation ✓ severe acute kidney injury after TAVI 1-year survival 59.3% in emergently patients 1-year survival 82.7% in elective patients	TAVI is a reasonable rescue therapy in patients with CS and decompensated AS.
Landes et al. [33]	Urgent TAVI in patients with severe AS and acute HF versus elective TAVI	High implantation success 96.3% Reduced 30-day functional capacity in urgent TAVI Similar 30-day mortality and MACE rates between urgent and elective TAVI	Urgent TAVI may be a viable treatment in patients with severe AS and acute decompensated HF, at high risk for surgery. Short-term outcomes after urgent TAVI appear to be reasonable.
Bongiovanni et al. [34]	Emergency TAVI versus emergency BAV followed by TAVI under elective circumstances in patients with severe AS	Immediate procedural mortality (eTAVI: 8.7%, vs. eBAV: 20.3%; *p* = 0.19) 30-day CV mortality (eTAVI: 23.8%, vs. eBAV: 33.0%; *p* = 0.40) Elective TAVI performed after eBAV → immediate procedural mortality 9.4% and 30-day CV mortality 15.6% Major vascular complications more likely to occur after eTAVI (*p* = 0.01) as well as stroke (*p* = 0.01)	Urgent TAVI is feasible in carefully selected patients with CS due to severe AS. High immediate procedural and 30-day mortality of eTAVI and eBAV. High mortality of secondary TAVI subsequent to eBAV.
Kolte et al. [18]	Patients with severe AS and CS undergoing urgent/emergent TAVI versus elective TAVI	Device success rate statistically lower, but not clinically different after emergent versus elective TAVI (92.6% vs. 93.7%; *p* = 0.007) Similar rates of major and/or life-threatening bleeding, major vascular complications, myocardial infarction, stroke, new permanent pacemaker placement, conversion to SAVR, and paravalvular regurgitation between groups Higher rates of acute kidney injury and/or new dialysis (8.2% vs. 4.2%; *p* < 0.001), 30-day mortality (8.7% vs. 4.3%, HR: 1.28, 95% CI: 1.10 to 1.48), and 1-year mortality (29.1% vs. 17.5%, HR: 1.20, 95% CI: 1.10 to 1.31) in emergent TAVI compared to elective Non-femoral access and cardiopulmonary bypass associated with increased risk in emergent TAVI Use of balloon-expandable valve associated with decreased risk of 30-day and 1-year mortality in emergent TAVI	Urgent/emergent TAVI is feasible with acceptable outcomes and a reasonable option in patients with severe AS.
Huang et al. [36]	Patients with decompensated severe AS and/or regurgitation and CS undergoing emergency TAVI	In-hospital mortality 19.4% 1-year survival rate 61.0% 2-year survival rate 55.9% Predictors of in-hospital mortality ✓ preprocedural pulmonary artery pulsatility index (PAPi) ≤ 1.8 (66.7% vs. 20.0%, *p* = 0.01) ✓ intraprocedural CPR (83.3% vs. 4.0%, *p* ≤ 0.001) ✓ acute kidney injury post-TAVI (80.0% vs. 4.2%, *p* ≤ 0.001) ✓ initiation of dialysis post-TAVI (60.0% vs. 4.2%, *p* ≤ 0.001) ✓ MCS initiation post-TAVI (50.0% vs. 12.0%, *p* = 0.03) MCS initiation before TAVI was associated with improved survival	Emergency TAVI in extreme risk patients with acute decompensated HF or CS due to severe aortic valve disease is associated with high in-hospital mortality rates. Right heart function and early utilization of MCS may improve survival following emergency TAVI.
Bandyopadhya et al. [37]	Patients with severe AS and CS undergoing urgent BAV versus urgent TAVI	No statistically significant difference in in-hospital mortality between the 2 groups (*p* = 0.29) Higher risk of permanent pacemaker (OR:17.00, 95% CI: 9.13–31.97; *p* < 0.001), major bleeding requiring transfusion (OR: 1.93, 95% CI: 1.60–2.30; *p* < 0.001), complete heart block (OR: 8.40, 95% CI: 5.10–14.01; *p* < 0.001), and vascular complications (OR: 3.20, 95% CI: 1.23–8.60; *p* = 0.02) in the TAVI group compared to BAV	The optimal choice between urgent TAVI versus urgent BAV followed by elective TAVI in acute decompensated AS is not clear
Fraccaro et al. [35]	Patients with severe AS and CS treated by TAVI	Device success 94.1% 30-day events ✓ mortality 11.8% ✓ stroke 2.0% ✓ vascular complications 5.9% ✓ acute kidney injury 34% 1-year mortality rate 25.7% HF readmission 8.6%	TAVI seems to be safe and feasible for patients with CS and severe AS at early and long-term follow-up.
Masha et al. [38]	Patients undergoing TAVI after presenting with CS versus high-risk patients without cardiogenic shock	CS was associated with higher 30-day mortality (19.1% vs. 4.9%) and higher rates of complications compared to non-CS High procedural success rates > 90% Absence of 30-day major complications not associated with a marked reduction in 30-day mortality Risk of death from acute CS before TAVI strongly related to the degree of shock pre-procedure	TAVI appears to be a viable treatment option for patients with AS and acute CS with high procedural success. Elevated risk of death, depending on the degree of pre-procedural shock.
Piriou et al. [39]	Rescue TAVI in patients with CS and severe aortic disease	30-day mortality 7.9% 1 year mortality 21.1% Complications ✓ acute kidney failure ✓ left bundle branch block ✓ pacemaker implantation 29% rehospitalization within 1 year Left bundle branch block → mortality risk factor	Rescue TAVI is safe and effective therapy with acceptable survivorship in patients with CS and severe aortic disease.
Steffen et al. [40]	Patients with acute HF due to severe AS undergoing emergent TAVI versus non-shock versus elective TAVI	90-day mortality → CS: 42.6%, vs. non-CS: 15.9%, vs. elective group: 5.3% (*p* < 0.01) Higher 30-day composite endpoint device failure in critically ill groups [CS: OR, 2.86 (1.43–5.36); non-CS: OR, 1.74 (1.09–2.69)] compared with elective patients 90-day mortality predictors: ✓ mechanical ventilation ✓ haemofiltration ✓ elevated C-reactive protein or bilirubin ✓ hypotension before TAVI	Increased 90-day mortality after TAVI in critically ill patients, but survivors have similar outcomes as elective patients.
Castelo et al. [41]	Patients with severe AS and CS undergoing urgent/emergent TAVI versus elective TAVI	Urgent TAVI associated with: ✓ higher mortality (25.3 vs. 15.1%, *p* = 0.043) ✓ 30-day CV mortality (17.5 vs. 4%, *p* = 0.001) ✓ life-threatening bleeding (11.5 vs. 4.1%, *p* = 0.018) ✓ vascular complications (11.5 vs. 4.6%, *p* = 0.031) ✓ longer hospital stay (28 vs. 12 days, *p* < 0.0001), compared to elective TAVI	Urgent TAVI showed worse short-term outcomes compared to elective TAVI. This may be due to worse baseline characteristics leading to the urgent nature of the procedure.
Goel et al. [42]	Patients with severe AS and CS treated by TAVI	Implantation success 97.9% Technical success 94.5% CS associated with higher in-hospital (9.9% vs. 2.7%), 30-day (12.9% vs. 4.9%), and 1-year (29.7% vs. 22.6%) mortality compared to the patients undergoing TAVI without CS Risk of 1-year mortality similar between groups [HR: 1.07, 95% CI: 0.95–1.21] Significant improvements in functional class (Class I/II 89%) and quality of life (ΔKCCQ score +50) at 1 year Older age (HR 1.02, 95% CI 1.02–1.03), peripheral artery disease (HR 1.25, 95% CI 1.06–1.47), prior implantation of an implantable cardioverter-defibrillator (HR 1.37, 95% CI 1.07–1.77), patients on dialysis (HR 2.07, 95% CI 1.69–2.53), immunocompromised status (HR 1.33, 95% CI 1.05–1.69), NYHA class III/IV (HR 1.50, 95% CI 1.06–2.12), lower aortic valve mean gradient, lower albumin levels, lower hemoglobin levels, and lower KCCQ scores independently associated with 1-year mortality	TAVI is safe and effective treatment for AS patients presenting with CS. Patients who survived the first 30 days after TAVI have similar mortality rates to those who were not in CS.
Nair et al. [19]	Patients with CS due to severe AS undergoing TAVI versus BAV versus medical therapy	Medical management associated with the highest 30-day mortality when compared with either balloon aortic valve replacement or aortic valve replacement (surgical or TAVI) (HR: 3.69 [95% CI: 2.04–6.66]; *p* < 0.0001) Higher 30-day mortality in BAV versus TAVI (26% versus 4%, *p* = 0.02) SAVR and TAVI showed lower 30-day and 1-year mortality than medical management (*p* < 0.05) and BAV (*p* < 0.05)	Higher in-hospital and 30-day mortality in CS due to severe AS compared with CS without AS. Urgent SAVR or TAVI associated with favorable short-and long-term outcomes. 50% mortality in BAV if not followed by definitive aortic valve replacement within 90 days.

TAVI, transcatheter aortic valve implantation; BAV, balloon aortic valvuloplasty; AS, aortic stenosis; HR, hazard ratio; CI, confidence interval; CPR, cardiopulmonary resuscitation; CS, cardiogenic shock; CV, cardiovascular; HF, heart failure; KCCQ, Kansas City Cardiomyopathy Questionnaire; MACE, major adverse cardiovascular events; MCS, mechanical circulatory support; NYHA, New York Heart Association; OR, odds ratio; PAPi, pulmonary artery pulsatility index; SAVR, surgical aortic valve replacement.4. Prognosis After TAVI in Patients with Severe Aortic Stenosis and Cardiogenic Shock.

## Abbreviations

AS	Aortic Stenosis
ACC	American College of Cardiology
ACS	Acute Coronary Syndrome.
BAV	Ballon Aortic Valvuloplasty
CAD	Coronary Artery Disease
CS	Cardiogenic Shock
CTCA	Computed Tomography Coronary Angiography
ECMO	Extracorporeal Membrane Oxygenation
IABP	Intra-Aortic Balloon Pump
KCCQ	Kansas City Cardiomyopathy Questionnaire
LV	Left Ventricle/Left Ventricular
MCS	Mechanical Circulatory Support
MACE	Major Adverse Cardiovascular Events
PCI	Percutaneous Coronary Intervention
TAVI	Transcatheter Aortic Valve Implantation
VA	Venoarterial
TV	Transvalvular
TA	Transapical
TOE	Transoesophageal Echocardiography
SAVR	Surgical Aortic Valve Replacement
STS	Society of Thoracic Surgeons
TVT	Transcatheter Valve Therapy
